# Blocking MyD88 signaling with MyD88 inhibitor prevents colitis-associated colorectal cancer development by maintaining colonic microbiota homeostasis

**DOI:** 10.1038/s41598-023-49457-8

**Published:** 2023-12-18

**Authors:** Bin Xie, Bo Wang, Runshi Shang, Lu Wang, Xia Huang, Lin Xie

**Affiliations:** 1 Institute of Organ Transplantation, Tongji Hospital, Tongji Medical College, Huazhong University of Science and Technology, Wuhan, 430030 China; 2 Key Laboratory of Organ Transplantation, Ministry of Education, Wuhan, China; 3 NHC Key Laboratory of Organ Transplantation, Wuhan, China; 4 Key Laboratory of Organ Transplantation, Chinese Academy of Medical Sciences, Wuhan, China; 5 Department of Gastroenterology, Tongji Hospital, Tongji Medical College, Huazhong University of Science and Technology, Wuhan, China

**Keywords:** Gastrointestinal cancer, Cancer, Microbiology

## Abstract

Certain intestinal microbiota alterations appear to positively correlate with tumorigenesis of CAC due to the disruption of the balance between the host and microorganisms. It is proven that blocking MyD88 signaling can prevent colitis-associated colorectal cancer (CAC) development in mice. We are aim to reveal the role of MyD88 signaling of maintaining colonic microbiota homeostasis for preventing CAC development. We here analyzed the landscape of gut microbiome in the mice model of AOM/DSS-induced CAC with MyD88 inhibitor treatment. PCoA revealed significant reduction in Lactobacillus load and increase in *Escherichia* load in the mucosal microbial composition of mice with CAC, compared with normal controls (NCs). Inhibitor-treatment led to almost undetectable *Proteobacteria (Escherichia)* and the retention of the dominance of *Firmicutes* and *Bacteroidota (Muribaculaceae)* in the mucosa. RNA sequencing analysis identified genes were up-regulated (Hp, SAA3 and IL-1F9) and down-regulated (CYP3A44, SLC30A10, GPNMB and OTC) in Inhibitor-treated mice (vs. CAC). Meanwhile, Inhibitor-treated mice had higher percentage of MUC2-positive area in colon sections (*vs*. CAC, which was less than NCs) by IF staining and decreased *Escherichia* in the mucus layer (vs. CAC) by FISH. And intestinal microbiota from mice with MyD88 inhibitor treatment could lessen the outcome of CAC by fecal microbiota transplantation. The development of CAC was involved in the increasing and ectopic *Escherichia* in the decreasing colonic mucus layer. MyD88 signaling blockade may maintain the host-microbiota homeostasis by up-regulating MUC2 production, increasing probiotics and their protective effects, and inhibiting the reproduction of *Escherichia*.

## Introduction

It is well known that the contribution of colitis and the immune system to carcinogenesis is a distinctive characteristic of colitis-associated colorectal cancer (CAC). Intestinal microbiota appears to be crucial to trigger the development of intestinal inflammation, which indicates a result of innate immune responses to Toll-like receptors (TLRs) activated by recognizing microbial colonization^1^. TLRs activation can induce intestinal microbiota dysbiosis and further increase the susceptibility to colitis and tumorigenesis^2^. There are reports demonstrated that TLRs activation by recognition of intestinal commensal microbes is influential for the regeneration of colonic epithelial cell upon dextran sodium sulfate (DSS) injury^3–5^. While the molecular mechanisms between intestinal microbes and CAC development have not been fully elucidated.

Myeloid differentiation factor 88 (MyD88) is one of the most important adapter molecules of TLRs, exerting biological abilities by activating downstream NF-κB^6^ and mitogen-activated protein kinase (MAPK)^7^ pathways, which initiate various pro-inflammatory responses. TLR/MyD88 signaling pathway plays a great role in maintaining intestinal microbiota homeostasis, participating in inflammatory responses, inducing tumor cell cycle arrest, and modulating the host immunity against colorectal cancer^8^. Our previous study has proven that the administration of a kind of novel MyD88 inhibitor, TJ-M2010-5, can completely prevent CAC development in mice by relieving inflammation and carcinogenesis^9^. Herein, we analyzed the landscape of gut microbiome in the mice model of AOM/DSS-induced CAC with MyD88 inhibitor administration to reveal the important role of MyD88 signaling of maintaining colonic microbiota homeostasis for preventing CAC development. Meanwhile, we further investigated the relieved effects of gut microbial community from MyD88 inhibitor-treated donor on CAC development, compared with the mice treated with fecal microbiota transplantation (FMT) and sterile fecal filtrate (SFF).

## Materials and methods

### Animals

6-week-old female BALB/c mice were obtained from GemPharmatech Co. (Nanjing, China) and kept under specific pathogen-free conditions. All animal protocols were approved by the Animal Care and Research Committee of Huazhong University of Science and Technology (Wuhan, China).

Mice were randomly separated into three groups (N = 3–6). (1) CAC group: mice were administrated with10 mg/kg AOM (Sigma-Aldrich Chemical) intraperitoneally (*i.p.*) and seven days later, they received three cycles of 2.5% DSS-drinking water (MP Biomedicals) for 1 week and 2 weeks of regular drinking water during a 10-week observation period; (2) MyD88 inhibitor (Inhibitor) group: AOM/DSS-induced mice were injected with 50 mg/kg MyD88 inhibitor (TJ-M2010-5, dissolved in sterile water) *i.p.* daily beginning 2 days before the first DSS administration during the 10-week-observation period; and (3) normal control (NC) group: gender- and age-matched healthy NC mice were raised in plain water and injected with sterile water.

### Ethics declarations

All animal protocols were approved by the Animal Care and Research Committee of Huazhong University of Science and Technology (Wuhan, China). All experiments were performed in accordance with the relevant guidelines and regulations within ARRIVE (Animal Research: Reporting of In Vivo Experiments) guidelines. Written informed consent for publication of this paper was obtained from the Tongji Hospital, Huazhong University of Science and Technology (No. TJH-201803001) and all authors.

### Sampling and histology

Euthanasia of all mice in every group was performed by cervical dislocation after anesthesia with 1.5% isoflurane at the end of the observation period. Mucosal samples in the distal part of the colon were collected and frozen at − 80 °C. Entire colons were opened longitudinally along its mesentery and flushed with ice-cold PBS. Tumor numbers were counted, and the average diameter of tumors was measured using a sliding caliper. Colons were fixed for 24 h in 10% formalin, embed in paraffin and prepared in 4 μm cross-sections. Sections were stained by hematoxylin–eosin (H&E) staining, and the severity of colitis was evaluated by pathological inflammatory scores of colons. The six-grade classification was: 0, structural change only; 1, chronic inflammation; 2, lamina propria neutrophils infiltration; 3, neutrophils in epithelium; 4, crypt destruction; and 5, erosions or ulcers. neoplasmas were identified in situ.

### DNA extraction

For the microbial community composition analysis, genomic DNA were extracted from mouse colon samples using the E.Z.N.A.® soil DNA Kit (Omega Bio-tek, USA) according to manufacturer’s instructions. The DNA extract was detected on 1% agarose gel. The concentration and purity of DNA were analyzed with NanoDrop 2000 UV–vis spectrophotometer (Thermo Scientific, USA).

### 16S rRNA amplicon sequencing and sequencing data analysis

The V3-V4 hypervariable region of the bacterial 16S rRNA gene was PCR-amplified with region-specific primers: 338F (5'-ACTCCTACGGGAGGCAGCAG-3') and 806R (5'-GGACTACHVGGGTWTCTAAT-3'). The purified amplicons were pooled in equimolar amounts, and paired-end sequencing was performed on an NovaSeq PE250 platform (Illumina, USA) according to the standard protocols by Majorbio Bio-Pharm Technology Co. (China).

The raw 16S rRNA gene sequencing reads were demultiplexed, quality-filtered by fastp (version 0.20.0)^10^ and merged by FLASH (version 1.2.7)^11^. Operational taxonomic units (OTUs) were clustered using UPARSE (version 7.1)^12^ with a 97% similarity cutoff, and chimeric sequences were identified and removed. Taxonomy was assigned to OUT representative sequence using RDP Classifier (version 2.2)^13^ against the Silva 138 database using confidence threshold of 0.7.

### RNA extraction and high throughput RNA sequencing (RNA-seq)

Total RNA was extracted from the colon tissue by TRIzol Reagent (Invitrogen, USA) and treated by DNase I (Takara, Japan) to remove genomic DNA. RNA-seq transcriptome libraries were prepared using the TruSeq RNA Sample Prep Kit (Illumina, USA) from 1 μg samples of total RNA. After quantification by using TBS380 fluorometer (Invitrogen, USA), the paired-end RNA-seq sequencing library was sequenced with an NovaSeq 6000 sequencer (2 × 150 bp read length) by Majorbio Bio-Pharm Technology Co. (China).

### Read mapping

Raw paired-end reads were trimmed and subjected to quality control by SeqPrep and Sickle with default parameters. The paired-end clean reads were mapped to the reference genome using HISAT2 with orientation mode^14^. The mapped reads of each sample were then assembled using StringTie in a reference-based approach^15^.

### Differential expression analysis (DEGs) and gene functional enrichment analysis

To identify DEGs between two different groups of samples, the expression level of each transcript was calculated according to the transcripts per million reads (TPM) method. RNA-seq by expected maximization (RSEM)^16^ was used to quantify gene expression levels. Virtually, the DESeq2^17^, DEGseq^18^ and EdgeR^19^ with Q-value ≤ 0.05 were used to analyze DEGs, which with |log2FC|> 1 and Q-value ≤ 0.05(DESeq2 or EdgeR) or Q-value ≤ 0.001(DEGseq) were considered to be differentially expressed. Moreover, GO functional enrichment analysis, carried out by Goatools, was performed to identify which DEGs were significantly enriched in GO terms, using a Bonferroni-corrected P-value ≤ 0.05.

### Immunohistochemistry (IHC), immunofluorescence (IF) and periodic acid Schiff (PAS) staining

Colon tissues of mice were fixed in Methanol–Carnoy’s fixative without washing, embedded in paraffin, and sectioned. Sections were dewaxed, hydrated, retrieved by RetrievagenA (BD Biosciences, USA). For IHC, sections were stained with anti-CD68 (Servicebio, China) antibodies. For IF, sections were stained with FITC-anti-MUC2, Cy3-anti-MPO, FITC-anti-CD4, Cy3-anti-CD8 (Abcam, UK) antibodies and DAPI (Servicebio, China). PAS staining was performed to visualize goblet cells and mucus layer in sections. Pictures were obtained by fluorescence microscope (Nikon Eclipse TI-SR, Japan) and CaseViewer2.4system (3DHISTECH, Hungary).

### Fluorescence in situ hybridization (FISH)

After being dewaxed and washed in 95% ethanol, colon tissue sections were hybridized overnight at 42 °C with 1μ MECO1167 (5-CY3-GCA TAA GCG TCG CTG CCG-3)/ BAC303 (5-CY5-CCA ATG TGG GGG ACC TT-3) probes. Cell nuclei were counterstained with DAPI, and images were acquired as above.

### FMT

In week 10, fresh feces of three groups (NC, CAC and Inhibitor) were pooled, homogenized with sterile saline and diluted to 50 mg feces/ml solution. Feces samples were centrifuged (100*g* × 2 min) and the supernatant was filtered through a 70 μm filters for FMT. For SFF, the supernatants were passed through 70 and 0.22 μm filters. Mice were subjected with polyethylene glycol (PEG, Servicebio, China, 200 μl at 425 g/L) by means of oral gavage every 20 min, 4 times in total, 4 h before the first FMT operation to depleting recipient microbiota^20^ and were administered with a total of 100 μl of FMT or SFF via oral gavage once a day beginning seven days before the AOM administration throughout a 10-week observation period.

### ELISA

Serum was prepared from the blood and analyzed for the presence of IL-6 and TNF-α (ebioscience) as the manufacturer’s suggested protocol.

### Statistical analysis

Statistical analysis between two groups was performed using the Student’s t test, or the Wilcoxon or the Kruskal–Wallis H test. Statistical analysis between more than two groups was performed using one-way analysis of variance (ANOVA). The normally distributed measurement data were presented as the Means ± SEM. A *P*-value of < 0.05 was considered to indicate a significant difference.

## Results

### Decreased alpha-diversity and altered mucosal microbiota in mice of CAC

**Figure 1:**
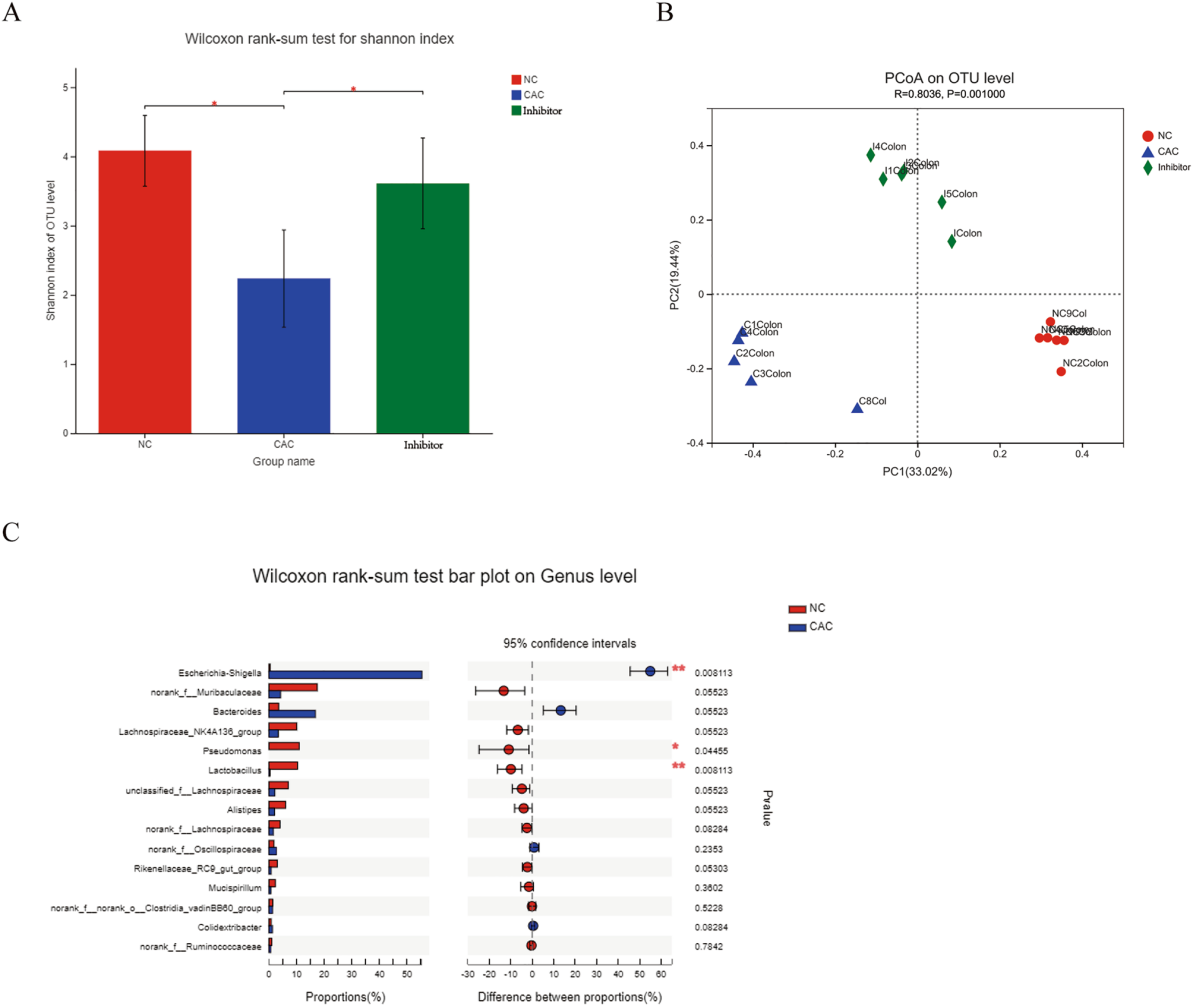
Comparisons of alpha-diversity and beta-diversity between normal controls (NC), CAC mice and Inhibitor-treated CAC mice. N = 6 per group. (**A**) Number of observed OTUs and Shannon diversity index were significantly reduced in CAC group compared with NC and Inhibitor groups. *P < 0.05. (**B**) PCoA of unweighted UniFrac analysisshowed that thecolonic mucosa microbiota composition was different between NC, CAC and Inhibitor groups (*P* = 0.001). (**C**) Comparison of proportions of gut microbiotaon the bacterial genus levels between NC and CAC groups. *P < 0.05, **P < 0.01.

In the AOM/DSS-induced CAC mouse model, CAC was strongly associated with a decrease in intra individual diversity, as measured by Shannon diversity index of single OTU, (*P* = 0.036 vs. NCs, Wilcoxon rank-sum test, Fig. 1A). Principal component analyses (PCoA) on OTU level and comparison of the abundance of single OTU displayed significant differences between the mucosal microbial composition of mice with CAC and that of controls (*P* = 0.001, Fig. 1B). Mice with CAC had an increased abundance of OTUs classified as *Escherichia* (*P* = 0.008) and a decreased abundance of OTUs classified as *Lactobacillus* (*P* = 0.008) and *Pseudomonas* (*P* = 0.04) in mucosa (Fig. 1C). These meant that the CAC burden mice had lower mucosal microbial alpha diversity and altered microbiome compared with normal control mice, especially, significant reduction in *Lactobacillus* load and increase in *Escherichia* load were involved in CAC tumorigenesis.

### MyD88 inhibitor treatment resulted in improvement of the composition of colonic microbiota in mice of CAC

TLR/MyD88 signaling pathway is critically involved in gastrointestinal epithelial cell homeostasis and tumorigenesis. Our patented MyD88 inhibitor, TJ-M2010-5, has been proved to be effective against MyD88 dimerization^9^. After its administration, it was shown that MyD88 signaling blockade in AOM/DSS-induced CAC mouse model led to complete suppression of CAC tumorigenesis. Here, it was demonstrated that gut microbiome alpha-diversity of Inhibitor-treated mice of CAC increased significantly, as measured by Shannon diversity index of single OTU (*P* = 0.013 vs. mice of CAC, Wilcoxon rank-sum test, Fig. 1A). PCoA on the single OTU level displayed significant differences among the mucosal microbial composition of CAC, Inhibitor-treated and NC mice (*P* = 0.001, Fig. 1B).

**Figure 2:**
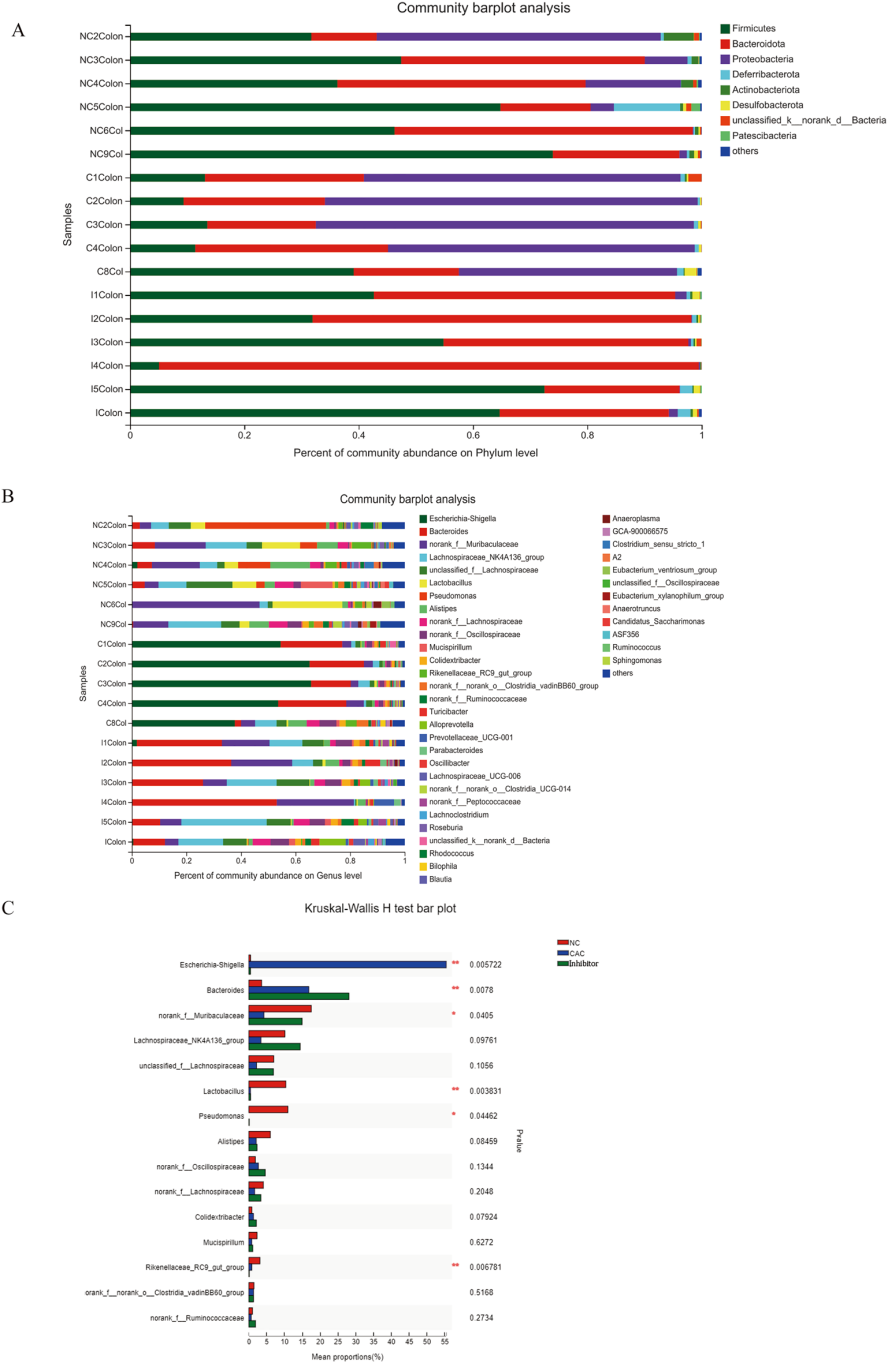
Taxonomic distribution of gut microbiota in mice from NC, CAC (C) and Inhibitor (I) groups. N = 6 per group. (**A**,**B**) Percentof community abundance of dominant gut microbiota among 3 groups on the phylum (**A**) and genus (**B**) levels. (**C**) Comparison of mean proportions of colonic mucosa microbiota on the bacterial genus levels among 3 groups. *P < 0.05, **P < 0.01.

To identify differentially abundant taxa, we performed community composition analysis on the mucosal microbiota composition of individuals from CAC (C), Inhibitor-treated (I) and NC groups. As shown in Fig. 2A, *Firmicutes* and *Bacteroidota* were the most dominant phylum in the colonic mucosa of NCs. And in the mucosa of carcinoma-bearing mice, significant increased *Proteobacteria* became dominant at the phylum level, with decreased percentage of *Firmicutes*. However, the MyD88 inhibitor treatment led to almost undetectable *Proteobacteria* in the mucosa and the retention of the dominance of *Firmicutes* and *Bacteroidota* at phylum level.

At the genus level, *Escherichia-Shigella* and *Bacteroides* were increased to dominance in the colonic mucosa of mice of CAC, along with less *norank_f_Muribaculaceae, Lachnospiraceae_NK4A136_group, unclassified_f_Lachnospiraceae, Lactobacillus, Pseudomonas, Alistipes*, etc. than the NCs. However, Inhibitor treatment restored the abundance of *norank_f_Muribaculaceae, Lachnospiraceae_NK4A136_group, unclassified_f_Lachnospiraceae*, etc. in the colonic mucosa, along with almost undetectable *Escherichia-Shigella* (Fig. 2B). The level of *Escherichia-Shigella* significantly raised in mice of CAC (*P* = 0.006 among the three groups, Kruskal–Wallis H test, Fig. 2C) and the level of *norank_f_Muribaculaceae* was significantly decreased in mice of CAC (*P* = 0.04 among the three groups). These meant MyD88 inhibitor preventing CAC development was associated with modulating of enteric microbiota dysbiosisduring colorectal tumorigenesis by decreasing *Escherichia, Proteobacteria* and increasing *Muribaculaceae, Bacteroidota*.

### MyD88 inhibitor treatment improved the microenvironment for colonic microbiota variations in mice of CAC

**Figure 3:**
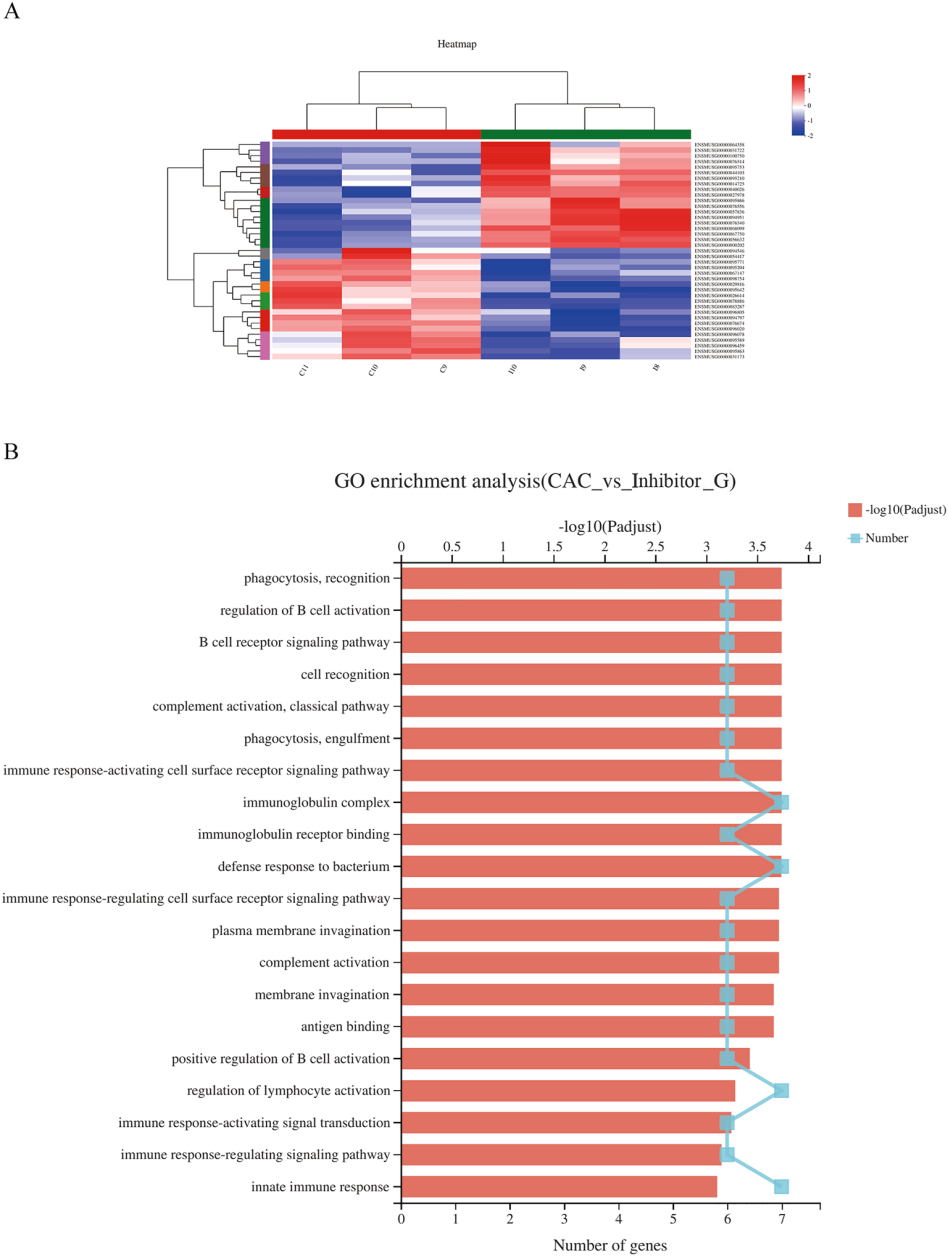
Effect of MyD88 inhibitor administration on the expression of genes associated with the microenvironment for colonic microbiota variations in mice with CAC. (**A**) Heatmap of RNA-seq analysis of colons from CAC and Inhibitor-treated CAC mice. N = 3 per group. (**B**) GO Enrichment analysis of differentially expressed genes of colons between CAC and Inhibitor-treated CAC groups.

Next, RNA-seq analysis was performed on colon tissue from Inhibitor-treated or untreated mice of CAC to investigate mechanisms by which MyD88 signaling-activation exaggerated colon tumorigenesis. Several genes with known roles in response to bacterium and immune response associated genes were significantly up-regulated [haptoglobin (Hp), serum amyloid antigen 3 (SAA3), interleukin 1 family, member 9 (IL-1F9)] and down-regulated[cytochrome P450, family 3, subfamily a, polypeptide 44(CYP3A44), solute carrier family 30 member 10 (SLC30A10), Glycoprotein Nonmetastatic Melanoma Protein B (GPNMB), ornithine transcarbamylase (OTC)] in the Inhibitor-treated mice compared with untreated mice of CAC (Fig. 3A,B, full gene list in Supplementary Material Table S1, GO Enrichment Analysis Statistics in Supplementary Material Table S2). Haptoglobin and SAA3 were well described for their antibacterial activity. Our results showed that the MyD88 signaling blockade might play an essential role in correcting enteric microbiota dysbiosis in colon tumors by up-regulating Hp and SAA3. Furthermore, IL-1F9 and CYP3A44 were involved in the innate and adaptive immune responses. SLC30A10 and GPNMB were both related to tumor-promoting inflammation and tumor progression. These data indicated that the MyD88 inhibitor improved the inflammatory and immune microenvironment for colonic microbiota variations, and further prevented tumor progression.

### MyD88 inhibitor treatment ameliorated gut barrier

**Figure 4:**
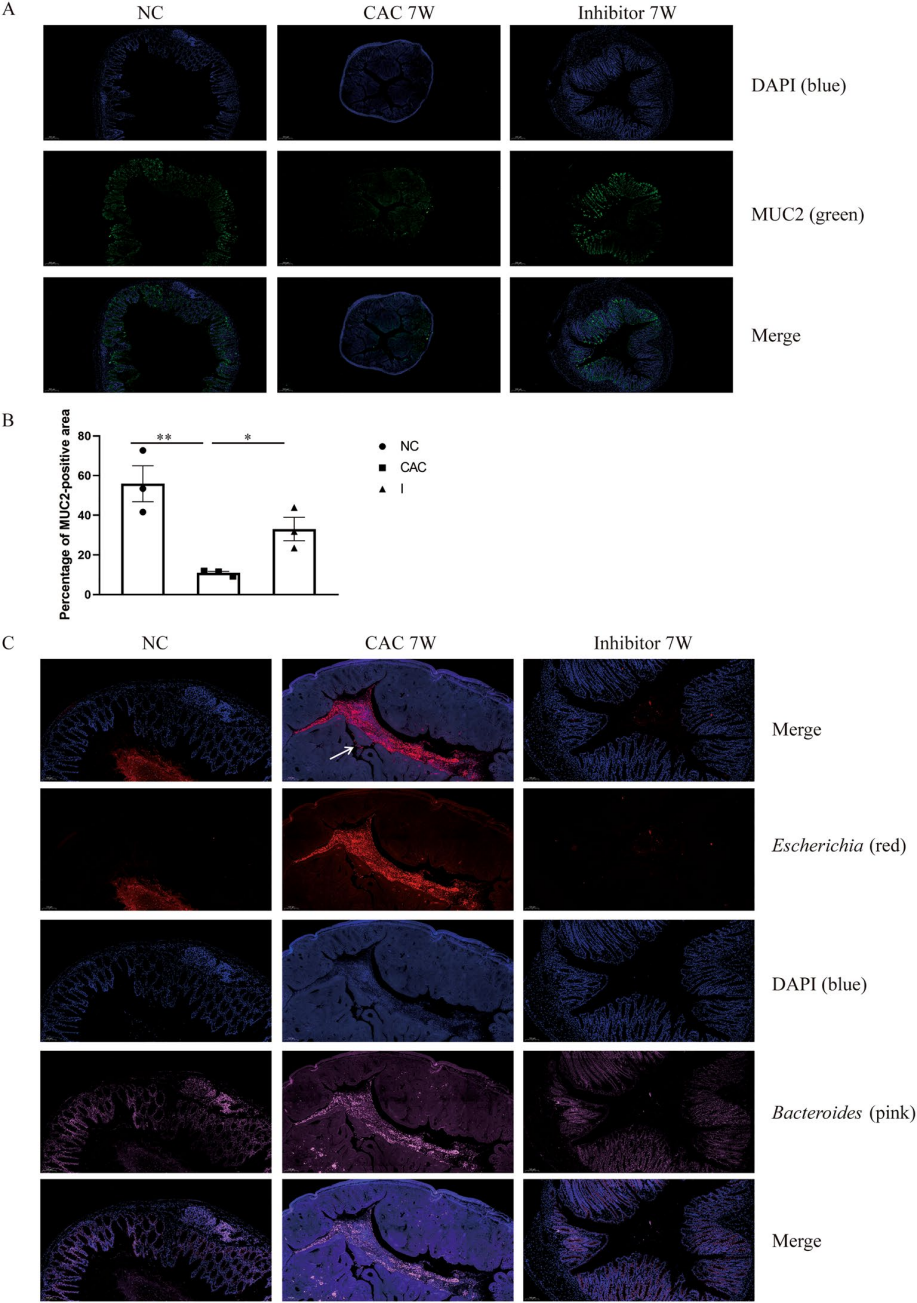
MUC2 secretion and the composition and position of bacteriain the mouse colon from NC, CAC and MyD88 inhibitor (Inhibitor)-treated groups. (**A**) IF staining of MUC2 (green) in the mouse colone remeals mucus-filled goblet cells in the epithelium and secreted mucus (nuclei stained with DAPI, blue). (**B**) Hitogram of the percentage of MUC2-positive area in the mouse colons of 3 groups. Values are Means ± SEM. N = 3 per group. *P < 0.05, **P < 0.01. (**C**) FISH using bacterial probes (red for *Escherichia* and pink for *Bacteroides*), and epithelial cells DNA stained with DAPI (blue) in colon sections. *Escherichia* DNA (red) and epithelial DNA (blue) are clearly separated in NC mice, but not in mice with CAC, in which *Escherichia* enter into the crypts and cells (white arrow). Unlike the CAC group, the dominant bacteria DNA staining was *Bacteroides* (pink) in the colons of NC and Inhibitor-treated mice.

The mucus layer overlying the mucosal epithelium, which comprises mucins and other materials, is a critical composition of the gut barrier. The major protein was mucin-2 (MUC2) that builds the colon mucus. It was reported that recombinant murine SAAs (rSAAs) induced enhancement of gut immune barrier by up-regulating MUC2 secretion, which provides the protection against bacterial invasion for colon mucosa^21^. Because we found that the MyD88 inhibitor treatment induced the up-expressing of SSA3 in the colon of mice of CAC, we here detected the alterations of colonic mucus area and the composition and the location of microbiota (*Escherichia* and *Bacteroides*) in the colon after the MyD88 inhibitor administration 7 weeks post-induction. IF staining of mouse colon sections with an anti-MUC2 Abs (green) identified the mucus layer and MUC2-positive goblet cells (Fig. 4A, nuclei stained with DAPI). As shown in Fig. 4B, the percentage of MUC2-positive area of Inhibitor-treated mouse colon with CAC was (33.1 ± 10.3) % and much more than that of untreated mice with CAC (10.9 ± 1.5) % (*P* = 0.021), which had significantly less mucus than NCs (55.9 ± 15.7) % (*P* = 0.008). In addition, we detected the colon sections for bacterial presence by FISH using a general 16S rRNA probe (Fig. 4C). *Escherichia* (red) and *Bacteroides* (pink) were detected in the mucus layer of colon. It was shown that there were lots of *Escherichia* (red) in the outer mucus layer of mouse with CAC, while the dominant bacteria were *Bacteroides* (pink) in the colons of NC and Inhibitor-treated mice. What’s more, *Escherichia* (red) were in direct contact with the colon epithelial cells and even deep down into the colonic crypts in mouse with CAC.

**Figure 5:**
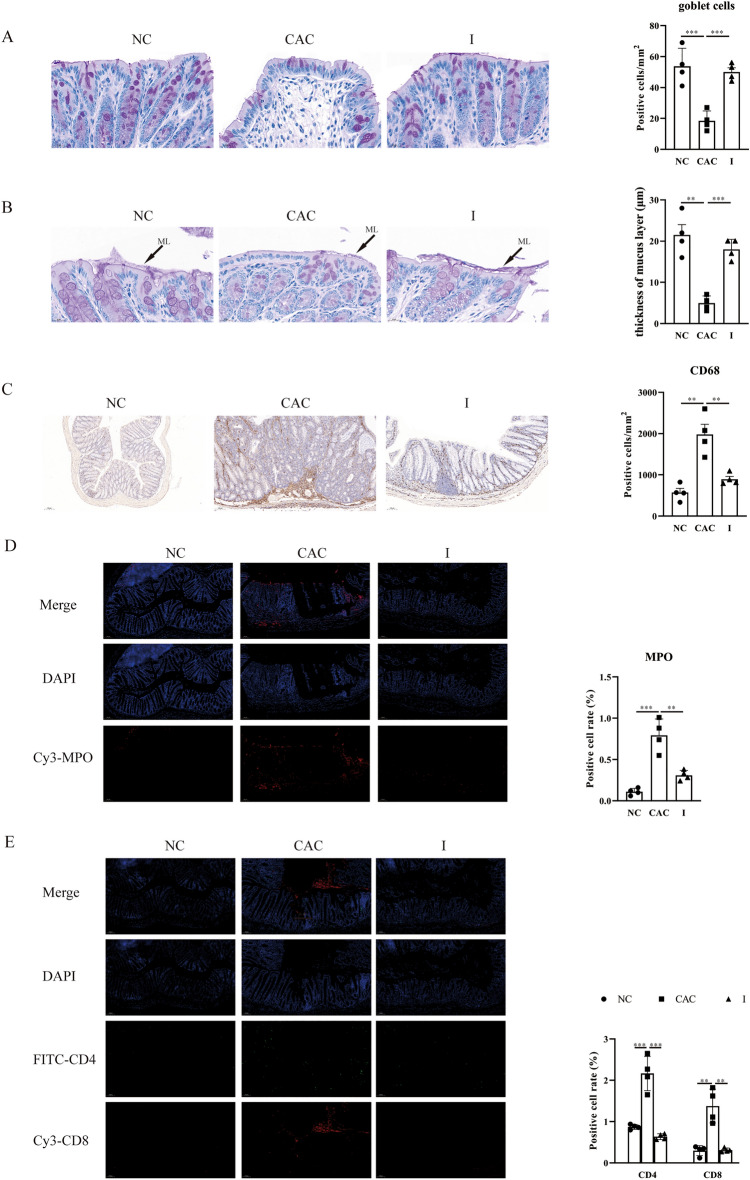
MyD88 inhibitor treatment ameliorated inflammatory cells infiltration. (**A**) The number of goblet cells per mm^2^ in the colon of mice stained with PAS staining. (**B**) The morphology of colonic mucosa of mice assessed by PAS staining. The arrows indicate the presence of mucus layer (ML). The thickness of mucus layer was shown in histogram. IHC staining for (**C**) CD68 and IF staining for Cy3-MPO (**D**) and FTIC-CD4/Cy5-CD8 (**E**) of colon sections. *NC* normal control group, *CAC* colitis-associated cancer group, *I* inhibitor-treated CAC group. N = 4 per group. All data are expressed as the means ± SEM of each group. **P < 0.01; ***P < 0.001.

Moreover, MyD88 Inhibitor-treated mice with CAC exhibited a 2.7-fold numbers of PAS^+^ goblet cells/mm^2^ (Fig. 5A) and a 3.6-fold thickness of mucin layers on the mucosal surface (Fig. 5B), as compared to untreated mice with CAC. Additionally, MPO, CD68 and CD4/CD8 positive cells were detected in colon of mice with CAC w/o MyD88 Inhibitor treatment to investigate neutrophils, macrophages, and T cells infiltration separately by IHC or IF. As compared to untreated mice with CAC, the levels of MPO^+^ neutrophils, CD68^+^ macrophages, CD4^+^ T cells and CD8^+^ T cells were all significantly reduced in the colon following Inhibitor treatment (*P* = 0.0032, *P* = 0.0054, *P* = 0.0003 and *P* = 0.0002, separately, Fig. 5C–E).

All these data manifested that the development of AOM/DSS-induced CAC was involved in the increasing and ectopic *Escherichia* in the decreasing colonic mucus layer, accompanied by the increasing inflammatory cells infiltration. MyD88 inhibitor treatment kept the gut barrier by up-regulating MUC2 production, improving microbiota dysbiosis, decreasing inflammatory cell infiltration, which was related to its protection function in the CAC development.

### Inhibitor-FMT contributed to alleviating colitis and tumor burden in the murine model of CAC

**Figure 6:**
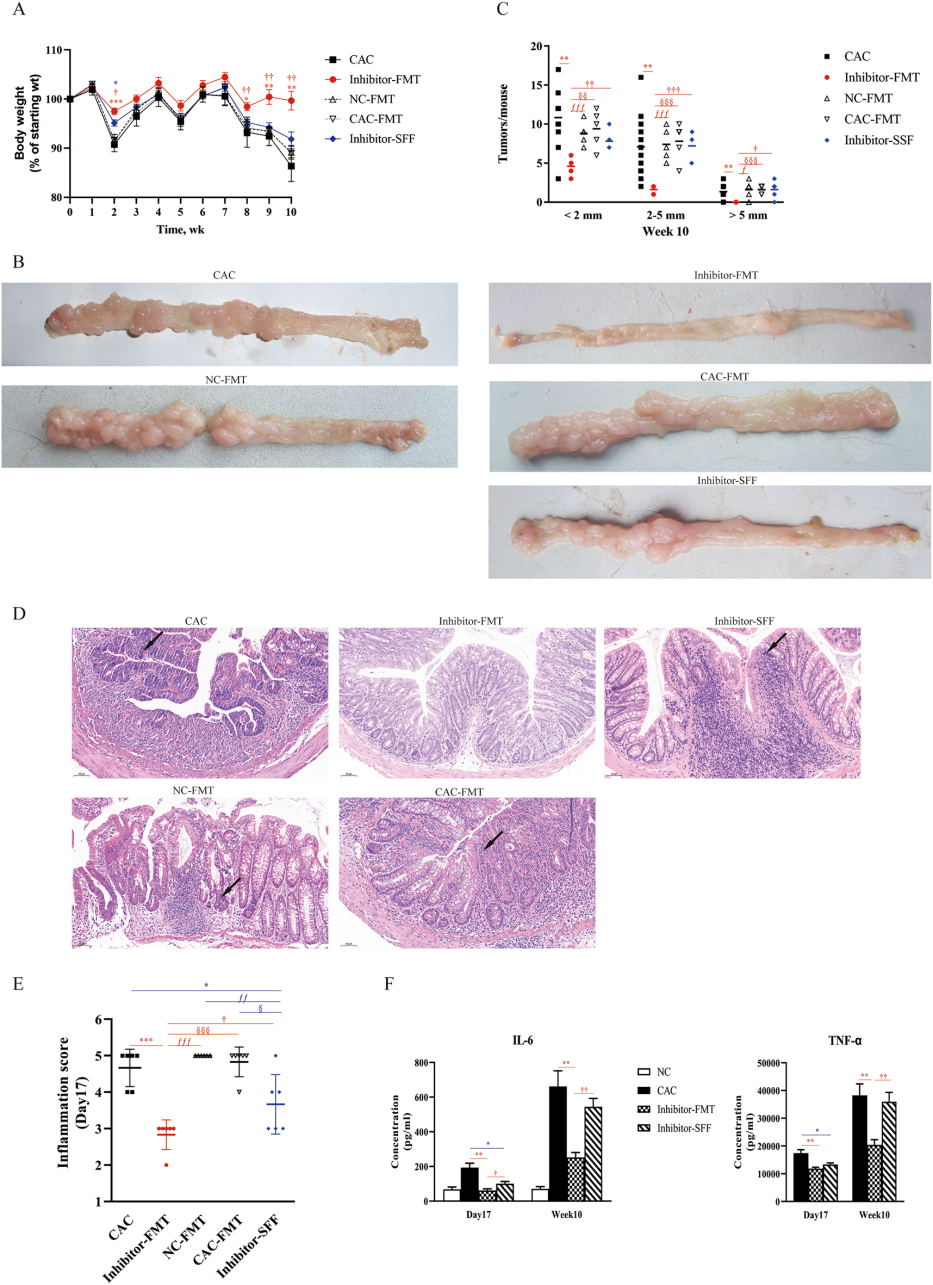
Inhibitor-FMT attenuated the symptoms of AOM/DSS-induced CAC. (**A**) Weekly body weight changes following AOM/DSS treatment. N = 10 ~ 20 per group. (**B**) Gross pathology and (**C**) size distribution of colorectal tumors of mouse colons at 10 weeks postinduction. N = 5 ~ 10 per group. (**D**) H&E staining of mouse colon sections and (**E**) histologic inflammation scores of colon tissues in mice at 17 days postinduction. Scale bar 50 μm. The arrows indicate the inflammatory cells infiltrating in the epithelium. N = 6 per group. (**F**) ELISA of IL-6 and TNF-α serum levels in mice at 17 days and 10 weeks postinduction. N = 4. All data are expressed as the Means ± SEM of each group. *P < 0.05; **P < 0.01; ***P < 0.001vs CAC. ^ƒ^*P* < 0.05; ^ƒƒ^*P* < 0.01; ^ƒƒƒ^*P* < 0.001vs NC-FMT. ^§^*P* < 0.05; ^§§^*P* < 0.01; ^§§§^*P* < 0.001vs CAC-FMT. ^†^*P* < 0.05; ^††^*P* < 0.01; ^†††^*P* < 0.001vs Inhibitor-SFF. Data from Inhibitor-FMT group is shown in red; data from Inhibitor-SFF group is shown in blue.

Consequently, we validated the impact of MyD88 inhibitor-mediated microbiota on CAC development via FMT derived from mice of CAC with MyD88 inhibitor treatment (Fig. 6). More increased body weight and less histological inflammatory damage and tumors were demonstrated in mice with CAC treated by Inhibitor-FMT compared with the four groups (CAC, NC-FMT, CAC-FMT, Inhibitor-SFF, Fig. 6A–E). As well, declined levels of IL-6 and TNF-α in the plasma were demonstrated in Fig. 6F in mice with CAC treated by Inhibitor-FMT (vs. CAC). Moreover, Inhibitor-SFF also alleviated the colitis in early inflammation (Day17) compared with mice with CAC, indicated by less body weight loss and histological score (Fig. 6A,E), while no significant differences in the colonic tumor burden of mice with CAC treated by Inhibitor-SFF were observed (vs. CAC) at the end of the 10-week observation period (Fig. 6B,C). The serum levels of IL-6 and TNF-α were decreased in early inflammation (Day 17) by Inhibitor-SFF compared with that of CAC, and showed no change at Week10 (Fig. 6F). Thus, blocking MyD88 signaling markedly influenced the composition of gut microbiome that contributed to alleviating colitis and tumor burden in the murine model of CAC.

## Discussion

It is well known that gut microbiota thrives in a mutually beneficial balance with the host. The host gut provides a nutrient-rich environment for located microbes, which in return help the host for food digestion, nutrient metabolism, providing various essential factors, protecting from pathogens and virus infection, and reshaping the host immune system^22^. Lots of reports have supported that certain bacterial species are associated with the initiation, development, progression of cancers, such as breast, cervical, lung, gastric, and colorectal cancers^23,24^.

This study confirms that the microbiota profile of colonic mucosa in CAC cases is distinct from healthy controls. In accordance with reported gut microbiome profiles, both CAC and control subjects were dominated by *Firmicutes, Bacteroidota* and *Proteobacteria* phylum with CAC being associated with different proportions of them^25^. Our analysis clearly underscores the disruption of enteric flora community in CAC with reduction in *Lactobacillus* load and increase in *Escherichia* load. It is proved that the colonic microbiota and microbial dysbiosis lead to colon tumorigenesis^26,27^. And it is demonstrated that there is significantly decreased colon tumorigenesis in germ-free mouse models^28^. Furthermore, *Escherichia coli* was directly associated with tumor activity^29^. Thus, reduction in *Escherichia* load in colonic mucosa and alternation of microenvironment suitable for the survival of *Escherichia* may enhance the therapeutic effect of anticancer drugs on CAC.

Our previous work clearly demonstrates blockade of MyD88 signaling with our novel Myd88 inhibitor, TJ-M2010-5, profoundly relieves colitis and tumor growth in the AOM/DSS-induced CAC mouse model^9^. The new findings of this study are that the blockade of MyD88 signaling pathway significantly improves the enteric microbiota dysbiosis during colorectal tumorigenesis by decreasing *Escherichia, Proteobacteria* and increasing *Firmicutes* and *Bacteroidota* in mice. It seems that the mechanisms by which our MyD88 inhibitor prevents CAC from developing are related to inhibiting the reproduction of *Escherichia* and increasing probiotics (*Muribaculaceae, Lactobacillus*, etc.) and their protective effects. Probiotics are healthy bacteria with no pathogenicity and are beneficial to the host by improving intestinal microbial balance^30^. It is reported that MyD88 signaling is involved in the protective effects of probiotics on the leukocyte recruitment in colitis^31^. Pre-treatment with probiotics ameliorates DSS-induced colitis, while is not effective in MyD88^−/−^ mice with colitis. However, some reports suggest that alterations to the microbiota in T-MyD88^−/−^ mice brings out increased gastrointestinal disease^32^. And Tregs in the gut can detect microbiota to promote mucosal tolerance in a MyD88-dependent manner. Meanwhile, Treg-specific MyD88 deficiency exacerbates intestinal inflammation^33^. Thus, it is systemically blocking MyD88 signaling pathway instead of lymphocyte-specific MyD88 deficiency that is beneficial to maintain colonic microbiota homeostasis and to prevent colitis and CAC development. Moreover, our results (Fig. 6) suggested that the intestinal microbiota from mice with MyD88 inhibitor treatment could lessen the outcome of CAC by FMT. And several reports have also proved that specific FMT from cancer patients can ameliorate the antitumor effects^34^. Collectively, MyD88 inhibitor-mediated microbial community alternative played a key role in alleviation of colitis, and further protected CAC carcinogenesis.

The mucus layer and intestinal epithelial cells together build a natural physiological barrier against pathogens^35^. The deficiency of mucus layer in MUC2-deficient mice causes impaired intestinal barrier function, even spontaneous inflammation in the gut^36–38^. It is reported that IL-1F9 (IL-36γ) exerts protective effects by improving the microenvironment for intestinal probiotics growth and promoting the secretion of colonic mucus^39,40^. Meanwhile, rSAAs induced enhancement of MUC2 production to improve the host innate immunity in the gut and to build a protective intestinal barrier against bacterial invasion^21^. Consistent with these reports, we observed that SAA3 and IL-1F9 mRNA expression were significantly up-regulated by the treatment of our MyD88 inhibitor in mice of CAC with reduced inflammation and suppressed tumor growth. And Fig. 4 also showed that MyD88 signaling blockade caused more colonic mucus secretion and improving microbiota dysbiosis with less *Escherichia* and more *Bacteroides* in the mucus layer by FISH. All the data prove that the MyD88 inhibitor prevents CAC development by maintaining colonic microbiota homeostasis.

MyD88 plays dual functional roles in colorectal carcinogenesis and the role in promotion or reduction of tumor development and growth depends on the colitis-associated cancer animal models used^8,41^. There are many reports have proved that MyD88 contributes to adenoma growth and progression and colon carcinogenesis^42–44^. And inhibition of the MyD88 signaling using the selective MyD88 inhibitory peptide dramatically suppresses growth of immunogenic tumor^45^. Our previous report has proved that the MyD88 inhibitor (TJ-M2010-5) can completely prevent AOM/DSS-induced CAC development in WT mice model^9^. While, some studies reported that the MyD88 signaling possesses the protective role in the malignant transformation of intestinal inflammatory conditions to colorectal cancer. MyD88^−/−^ mice are more susceptible to AOM/DSS-induced colitis and form more polyps and have histological characteristics of more aggressive adenomas and carcinomas^46^. This result seems to be contradictory compared with our report. It is possibly because that the immune system and the immune response to inflammation and malignant transformation of tumors between the WT and the MyD88 deficiency mice are very distinctive. MyD88 knockout mice will undergo genomic defects and many compensatory physiological changes during embryonic development and growth to maintain survival. Current research demonstrated that the susceptibility to AOM/DSS-induced CAC in MyD88^−/−^ mice is at least in part due to an inability to signal through the IL-18R. While in the CAC model of WT mice with the MyD88 inhibitor treatment, we do not found lacking molecules associated with IL-18 signal transduction. Mechanisms of blockage of MyD88 signaling with the MyD88 inhibitor in preventing CAC development in mice need further investigation, and our studies showed that it is involved in reduced colitis and improved tumor immune microenvironment^47^.

### Supplementary Information

Supplementary Table 1.

Supplementary Table 2.

## Data Availability

The authors confirm that the data supporting the findings of this study are available within the article and its supplementary materials, and from the corresponding author, [lx], upon reasonable request.
