# Supplementary material for: Blocking MyD88 signaling with MyD88 inhibitor prevents colitis-associated colorectal cancer development by maintaining colonic microbiota homeostasis
Source: Sci Rep. 2023 Dec 18;13:22552. doi: 10.1038/s41598-023-49457-8 (PMC10728211; doi:10.1038/s41598-023-49457-8)
Supplement: Supplementary file 1 — Supplementary Table 1. [file 41598_2023_49457_MOESM1_ESM.pdf]

Gene_id	Gene name	Gene description	FC(I/CAC)	Log2FC(I/CAC)	Pvalue	Padjust	Signi-ficant	Regu-late	C10	C11	C9	I10	I9	I8	CAC	I
ENSMUSG00000094797	Igkv6-15	immunoglobulin kappa variable 6-15 [Source:MGI Symbol;Acc:MGI:1330831]	0.254	-1.978	2.16E-11	3.48E-07	yes	down	290.08	273.46	182.24	86.15	40.4	70.085	248.59	65.55
ENSMUSG00000094951	Ighv5-6	immunoglobulin heavy variable 5-6 [Source:MGI Symbol;Acc:MGI:4439815]	5.713	2.514	2.94E-09	6.77E-06	yes	up	27.82	18.45	42.39	112.22	231.9	180.835	29.55	174.99
ENSMUSG00000076674	Ighv3-8	immunoglobulin heavy variable V3-8 [Source:MGI Symbol;Acc:MGI:3645298]	0.308	-1.699	3.46E-09	6.96E-06	yes	down	68.96	61.84	70.68	26.33	12.95	24.26	67.16	21.18
ENSMUSG00000095771	Igkv14-111	immunoglobulin kappa variable 14-111 [Source:MGI Symbol;Acc:MGI:44	0.364	-1.456	2.68E-08	3.53E-05	yes	down	249.6	225.48	163.74	61.72	88.32	88.505	212.94	79.52
ENSMUSG00000029816	Gpnmb	glycoprotein (transmembrane) nmb [Source:MGI Symbol;Acc:MGI:1934765]	0.494	-1.018	8.28E-08	7.68E-05	yes	down	24.03	30.73	22.38	15.22	9.04	13.87	25.71	12.71
ENSMUSG00000078886	Gm2026	predicted gene 2026 [Source:MGI Symbol;Acc:MGI:3780195]	0.003	-8.268	1.37E-07	1.10E-04	yes	down	0.98	3.22	2.07	0	0	0	2.09	0.00
ENSMUSG00000040026	Saa3	serum amyloid A 3 [Source:MGI Symbol;Acc:MGI:98223]	2.835	1.503	2.71E-07	2.08E-04	yes	up	20.55	33.01	47.24	108.04	83.11	108.895	33.60	100.02

ENSMUSG00000068099	1500009C09Rik	RIKEN cDNA 1500009C09 gene [Source:MGI Symbol;Acc:MGI:1923755]	3.671	1.876	3.28E-07	2.28E-04	yes	up	1.19	1.08	1.8	5.28	4.36	5.21	1.36	4.95
ENSMUSG00000100750	Gm29084	predicted gene 29084 [Source:MGI Symbol;Acc:MGI:5579790]	2.086	1.061	6.14E-07	3.41E-04	yes	up	74.92	63.55	62.87	182.05	89.69	145.89	67.11	139.21
ENSMUSG00000076556	Igkv4-57	immunoglobulin kappa variable 4-57 [Source:MGI Symbol;Acc:MGI:2685035]	2.703	1.435	8.56E-07	4.44E-04	yes	up	167.74	111.09	181.73	292.73	532.9	425.64	153.52	417.09
ENSMUSG00000057836	Xlr3a	X-linked lymphocyte-regulated 3A [Source:MGI Symbol;Acc:MGI:109506]	8.701	3.121	1.52E-06	7.06E-04	yes	up	1.58	0.12	1.27	3.84	4.9	7.535	0.99	5.43
ENSMUSG00000031722	Hp	haptoglobin [Source:MGI Symbol;Acc:MGI:96211]	2.883	1.528	1.89E-06	8.23E-04	yes	up	4.83	4.68	5.69	19.77	4.58	19.42	5.07	14.59
ENSMUSG00000095210	Ighv5-9-1	immunoglobulin heavy variable 5-9-1 [Source:MGI Symbol;Acc:MGI:4439810]	5.162	2.368	2.11E-06	8.35E-04	yes	up	21.31	7.73	17.34	120.57	33.14	91.3	15.46	81.67
ENSMUSG00000095642	Ighv14-3	immunoglobulin heavy variable V14-3 [Source:MGI Symbol;Acc:MGI:4439764]	0.486	-1.040	2.56E-06	9.82E-04	yes	down	273.96	428.5	291.4	191.03	131.57	170.6	331.29	164.40

ENSMUSG00000095866	Ighv2-4	immunoglobulin heavy variable V2-4 [Source:MGI Symbol;Acc:MGI:3643263]	7.856	2.974	3.06E-06	1.12E-03	yes	up	6.24	6.07	4.42	20.4	68.33	47.74	5.58	45.49
ENSMUSG00000095863	Ighv1-67	immunoglobulin heavy variable V1-67 [Source:MGI Symbol;Acc:MGI:3645228]	0.22	-2.186	4.34E-06	1.45E-03	yes	down	67.29	35.91	94.43	16.05	11	16.285	65.88	14.45
ENSMUSG00000095589	Ighv1-55	immunoglobulin heavy variable 1-55 [Source:MGI Symbol;Acc:MGI:4439716]	0.239	-2.064	5.15E-06	1.69E-03	yes	down	124.78	38.85	104.44	20.11	20.02	25.87	89.36	22.00
ENSMUSG00000044103	Il1f9	interleukin 1 family, member 9 [Source:MGI Symbol;Acc:MGI:2449929]	2.704	1.435	6.79E-06	2.13E-03	yes	up	4.64	2.45	2.57	8.94	6.53	10.195	3.22	8.56
ENSMUSG00000094546	Ighv1-26	immunoglobulin heavy variable 1-26 [Source:MGI Symbol;Acc:MGI:4439641]	0.485	-1.044	1.10E-05	3.22E-03	yes	down	267.08	115.02	148.59	147.78	106.39	138.425	176.90	130.87
ENSMUSG00000096459	Ighv9-3	immunoglobulin heavy variable V9-3 [Source:MGI Symbol;Acc:MGI:3642720]	0.402	-1.313	1.13E-05	3.24E-03	yes	down	190.71	101.16	167.32	62.14	64.11	65.42	153.06	63.89

ENSMUSG00000076514	Igkv17-121	immunoglobulin kappa variable 17-121 [Source:MGI Symbol;Acc:MGI:3647671]	7.574	2.921	1.20E-05	3.39E-03	yes	up	13.26	6.65	12.66	150.58	17.38	92.5	10.86	86.82
ENSMUSG00000067750	Khdc1a	KH domain containing 1A [Source:MGI Symbol;Acc:MGI:2676610]	2.194	1.134	1.23E-05	3.42E-03	yes	up	6.18	4.58	7.94	12.51	10.48	17.51	6.23	13.50
ENSMUSG00000027978	Prss12	protease, serine 12 neurotrypsin (motopsin) [Source:MGI Symbol;Acc:MGI:1100881]	2.143	1.099	2.42E-05	5.85E-03	yes	up	3.95	5.97	7.46	12.77	9	15.085	5.79	12.29
ENSMUSG00000056632	Dsg3	desmoglein 3 [Source:MGI Symbol;Acc:MGI:99499]	2.027	1.020	2.80E-05	6.21E-03	yes	up	1.98	1.64	2.08	3.34	3	4.46	1.90	3.60
ENSMUSG00000067147	Rpl7a-ps11	ribosomal protein L7A, pseudogene 11 [Source:MGI Symbol;Acc:MGI:3644020]	0.373	-1.422	2.82E-05	6.21E-03	yes	down	10.75	10.81	9.85	3.04	3.94	4.705	10.47	3.90
ENSMUSG00000096078	Ighv1-62-2	immunoglobulin heavy variable 1-62-2 [Source:MGI Symbol;Acc:MGI:3644968]	0.181	-2.470	5.10E-05	9.66E-03	yes	down	69.64	18.48	43.67	4.7	9.72	10.045	43.93	8.16

ENSMUSG00000095753	Igkv4-53	immunoglobulin kappa variable 4-53 [Source:MGI Symbol;Acc:MGI:2686266]	4.472	2.161	6.69E-05	1.20E-02	yes	up	96.06	116.48	71.38	683.29	273.17	493.2	94.64	483.22
ENSMUSG00000031173	Otc	ornithine transcarbamylase [Source:MGI Symbol;Acc:MGI:97448]	0.406	-1.300	8.44E-05	1.35E-02	yes	down	3.1	2.16	2.76	1.09	0.96	1.16	2.67	1.07
ENSMUSG00000014725	Adam28	a disintegrin and metallopeptidase domain 28 [Source:MGI Symbol;Acc:MGI:105988]	3.889	1.959	1.19E-04	1.65E-02	yes	up	2.34	0.68	1.16	6.37	2.06	6.465	1.39	4.97
ENSMUSG00000076540	Igkv4-80	immunoglobulin kappa variable 4-80 [Source:MGI Symbol;Acc:MGI:4439653]	7.753	2.955	1.20E-04	1.65E-02	yes	up	38.23	37.44	93.9	249.49	654.35	464.19	56.52	456.01
ENSMUSG00000064358	mt-Co3	mitochondrially encoded cytochrome c oxidase III [Source:MGI Symbol;Acc:MGI:102502]	209.5	7.711	1.37E-04	1.82E-02	yes	up	0.29	0.29	0.29	125.86	0.27	63.08	0.29	63.07
ENSMUSG00000083287	Idi1-ps1	isopentenyl-diphosphate delta isomerase, pseudogene 1 [Source:MGI Symbol;Acc:MGI:3649894]	0.204	-2.297	1.93E-04	2.46E-02	yes	down	3.43	5.92	4.4	1.06	0.52	1.24	4.58	0.94

ENSMUSG00000096020	Ighv1-75	immunoglobulin heavy variable 1-75 [Source:MGI Symbol;Acc:MGI:4439735]	0.181	-2.465	1.94E-04	2.46E-02	yes	down	18.39	13.22	12.36	3.47	1.82	2.855	14.66	2.72
ENSMUSG00000096805	Ighv9-1	immunoglobulin heavy variable 9-1 [Source:MGI Symbol;Acc:MGI:4439911]	0.464	-1.108	1.96E-04	2.46E-02	yes	down	159.52	101.35	115.88	79.12	33.88	63.07	125.58	58.69
ENSMUSG00000095204	Ighv1-52	immunoglobulin heavy variable 1-52 [Source:MGI Symbol;Acc:MGI:4439752]	0.289	-1.792	2.13E-04	2.52E-02	yes	down	41.56	43.84	22.87	6.99	11.43	14.13	36.09	10.85
ENSMUSG00000026614	Slc30a10	solute carrier family 30, member 10 [Source:MGI Symbol;Acc:MGI:2685058]	0.322	-1.635	2.71E-04	3.03E-02	yes	down	0.88	1.58	0.87	0.21	0.45	0.36	1.11	0.34
ENSMUSG00000000202	Btbd17	BTB (POZ) domain containing 17 [Source:MGI Symbol;Acc:MGI:1919264]	2.812	1.491	2.84E-04	3.11E-02	yes	up	1.03	0.68	1.04	2.44	2.57	2.565	0.92	2.53
ENSMUSG00000098754	Prn	prion protein readthrough transcript [Source:MGI Symbol;Acc:MGI:97767]	0.259	-1.947	3.31E-04	3.43E-02	yes	down	1.24	1.02	0.95	0.2	0.27	0.325	1.07	0.27

ENSMUSG00000054417	Cyp3a44	cytochrome P450, family 3, subfamily a, polypeptide 44 [Source:MGI Symbol;Acc:MGI:2449818]	0.101	-3.306	5.18E-04	4.73E-02	yes	down	2.89	0.29	1.48	0.25	0	0.215	1.55	0.16
