# Supplementary material for: Blocking MyD88 signaling with MyD88 inhibitor prevents colitis-associated colorectal cancer development by maintaining colonic microbiota homeostasis
Source: Sci Rep. 2023 Dec 18;13:22552. doi: 10.1038/s41598-023-49457-8 (PMC10728211; doi:10.1038/s41598-023-49457-8)
Supplement: Supplementary file 2 — Supplementary Table 2. [file 41598_2023_49457_MOESM2_ESM.pdf]

Number	GO ID	Term Type	Description	Ratio_in_study	Ratio_in_pop	Pvalue	Padjust
6	GO:0006910	BP	phagocytosis, recognition	6/28	106/34644	2.26531E-08	0.000181618
6	GO:0050864	BP	regulation of B cell activation	6/28	234/34644	2.96272E-08	0.000181618
6	GO:0050853	BP	B cell receptor signaling pathway	6/28	121/34644	4.40691E-08	0.000181618
6	GO:0008037	BP	cell recognition	6/28	261/34644	5.65921E-08	0.000181618
6	GO:0006958	BP	complement activation, classical pathway	6/28	128/34644	5.84231E-08	0.000181618
6	GO:0006911	BP	phagocytosis, engulfment	6/28	134/34644	7.34801E-08	0.000181618
6	GO:0002429	BP	immune response-activating cell surface receptor signaling pathway	6/28	273/34644	7.38164E-08	0.000181618
7	GO:0019814	CC	immunoglobulin complex	7/28	135/34644	7.6264E-08	0.000181618
6	GO:0034987	MF	immunoglobulin receptor binding	6/28	100/34644	1.68853E-08	0.000181618
7	GO:0042742	BP	defense response to bacterium	7/28	479/34644	8.51611E-08	0.000182526
6	GO:0002768	BP	immune response-regulating cell surface receptor signaling pathway	6/28	288/34644	1.01212E-07	0.000192386
6	GO:0099024	BP	plasma membrane invagination	6/28	147/34644	1.1669E-07	0.000192386
6	GO:0006956	BP	complement activation	6/28	147/34644	1.1669E-07	0.000192386
6	GO:0010324	BP	membrane invagination	6/28	153/34644	1.42443E-07	0.000217134
6	GO:0003823	MF	antigen binding	6/28	155/34644	1.51962E-07	0.000217134
6	GO:0050871	BP	positive regulation of B cell activation	6/28	175/34644	2.77789E-07	0.000372115
7	GO:0051249	BP	regulation of lymphocyte activation	7/28	605/34644	4.1198E-07	0.00051941
6	GO:0002757	BP	immune response-activating signal transduction	6/28	375/34644	4.76106E-07	0.00056691
6	GO:0002764	BP	immune response-regulating signaling pathway	6/28	393/34644	6.25743E-07	0.000705871
7	GO:0045087	BP	innate immune response	7/28	658/34644	7.2285E-07	0.000774642
6	GO:0051251	BP	positive regulation of lymphocyte activation	6/28	410/34644	8.00563E-07	0.00081707
6	GO:0042571	CC	immunoglobulin complex, circulating	6/28	93/34644	9.48224E-07	0.000923786
6	GO:0050851	BP	antigen receptor-mediated signaling pathway	6/28	228/34644	1.02635E-06	0.000947673
8	GO:0009617	BP	response to bacterium	8/28	697/34644	1.06117E-06	0.000947673
7	GO:0002694	BP	regulation of leukocyte activation	7/28	719/34644	1.30476E-06	0.0011186
7	GO:0050865	BP	regulation of cell activation	7/28	761/34644	1.9012E-06	0.001567244
6	GO:0002696	BP	positive regulation of leukocyte activation	6/28	481/34644	2.01853E-06	0.001602342
6	GO:0050867	BP	positive regulation of cell activation	6/28	497/34644	2.43744E-06	0.001865776
9	GO:0006955	BP	immune response	9/28	1624/34644	3.28366E-06	0.002426849

6	GO:0002253	BP	activation of immune response	6/28	553/34644	4.49932E-06	0.003214465
9	GO:0051707	BP	response to other organism	9/28	1708/34644	4.95785E-06	0.003427795
6	GO:0009897	CC	external side of plasma membrane	6/28	574/34644	5.56798E-06	0.003729328
8	GO:0098542	BP	defense response to other organism	8/28	1336/34644	7.46826E-06	0.004850521
9	GO:0006952	BP	defense response	9/28	1826/34644	8.52591E-06	0.005374586
11	GO:0044421	CC	extracellular region part	11/28	2956/34644	9.33653E-06	0.005717426
6	GO:0006959	BP	humoral immune response	6/28	694/34644	1.63583E-05	0.009739087
9	GO:0007166	BP	cell surface receptor signaling pathway	9/28	1998/34644	1.75767E-05	0.010073367
9	GO:0043207	BP	response to external biotic stimulus	9/28	2002/34644	1.78597E-05	0.010073367
9	GO:0009607	BP	response to biotic stimulus	9/28	2023/34644	1.94109E-05	0.010667523
6	GO:0061024	BP	membrane organization	6/28	726/34644	2.10852E-05	0.011297969
6	GO:0098552	CC	side of membrane	6/28	733/34644	2.22534E-05	0.011633095
10	GO:0002376	BP	immune system process	10/28	2660/34644	2.5312E-05	0.01291698
6	GO:0002252	BP	immune effector process	6/28	760/34644	2.72598E-05	0.013587424
10	GO:0009605	BP	response to external stimulus	10/28	2981/34644	6.74125E-05	0.032837566
6	GO:0050778	BP	positive regulation of immune response	6/28	970/34644	0.000105416	0.050208361
10	GO:0051704	BP	multi-organism process	10/28	3212/34644	0.000126629	0.059000652
9	GO:0048584	BP	positive regulation of response to stimulus	9/28	2790/34644	0.000236417	0.105933189
8	GO:0005102	MF	signaling receptor binding	8/28	2174/34644	0.000237241	0.105933189
6	GO:0050776	BP	regulation of immune response	6/28	1231/34644	0.000382512	0.167313899
15	GO:0050896	BP	response to stimulus	15/28	7911/34644	0.00039883	0.170962592
6	GO:0002684	BP	positive regulation of immune system process	6/28	1345/34644	0.00061161	0.257032022
7	GO:0002682	BP	regulation of immune system process	7/28	1905/34644	0.000640251	0.263894324
2	GO:0006953	BP	acute-phase response	2/28	51/34644	0.000783698	0.303905185
1	GO:0006593	BP	ornithine catabolic process	1/28	1/34644	0.000808221	0.303905185
1	GO:0070781	BP	response to biotin	1/28	1/34644	0.000808221	0.303905185
1	GO:2000296	BP	negative regulation of hydrogen peroxide catabolic process	1/28	1/34644	0.000808221	0.303905185
1	GO:0004585	MF	ornithine carbamoyltransferase activity	1/28	1/34644	0.000808221	0.303905185
1	GO:0097272	BP	ammonia homeostasis	1/28	2/34644	0.001615812	0.558575649
1	GO:0042450	BP	arginine biosynthetic process via ornithine	1/28	2/34644	0.001615812	0.558575649
1	GO:1905802	BP	regulation of cellular response to manganese ion	1/28	2/34644	0.001615812	0.558575649

9	GO:0044459	CC	plasma membrane part	9/28	3606/34644	0.001551499	0.558575649
1	GO:0016743	MF	carboxyl- or carbamoyltransferase activity	1/28	2/34644	0.001615812	0.558575649
2	GO:2000378	BP	negative regulation of reactive oxygen species metabolic process	2/28	82/34644	0.002009958	0.683800467
2	GO:0002377	BP	immunoglobulin production	2/28	88/34644	0.002309885	0.773558818
1	GO:0019240	BP	citrulline biosynthetic process	1/28	3/34644	0.002422773	0.775034242
1	GO:2000295	BP	regulation of hydrogen peroxide catabolic process	1/28	3/34644	0.002422773	0.775034242
1	GO:0005914	CC	spot adherens junction	1/28	3/34644	0.002422773	0.775034242
2	GO:0002526	BP	acute inflammatory response	2/28	101/34644	0.003027575	0.954265016
1	GO:1901740	BP	negative regulation of myoblast fusion	1/28	4/34644	0.003229105	0.988705965
1	GO:0030492	MF	hemoglobin binding	1/28	4/34644	0.003229105	0.988705965
1	GO:0071579	BP	regulation of zinc ion transport	1/28	5/34644	0.004034809	1
1	GO:0010727	BP	negative regulation of hydrogen peroxide metabolic process	1/28	5/34644	0.004034809	1
1	GO:0051195	BP	negative regulation of cofactor metabolic process	1/28	5/34644	0.004034809	1
1	GO:0071421	BP	manganese ion transmembrane transport	1/28	5/34644	0.004034809	1
1	GO:0000052	BP	citrulline metabolic process	1/28	6/34644	0.004839885	1
1	GO:0006526	BP	arginine biosynthetic process	1/28	6/34644	0.004839885	1
2	GO:0002440	BP	production of molecular mediator of immune response	2/28	132/34644	0.005104024	1
2	GO:0033273	BP	response to vitamin	2/28	136/34644	0.005408484	1
1	GO:0034242	BP	negative regulation of syncytium formation by plasma membrane fusion	1/28	7/34644	0.005644334	1
9	GO:0006950	BP	response to stress	9/28	4435/34644	0.006416727	1
1	GO:0033590	BP	response to cobalamin	1/28	8/34644	0.006448155	1
1	GO:0050993	BP	dimethylallyl diphosphate metabolic process	1/28	9/34644	0.00725135	1
1	GO:0050992	BP	dimethylallyl diphosphate biosynthetic process	1/28	9/34644	0.00725135	1
3	GO:0006954	BP	inflammatory response	3/28	502/34644	0.007569978	1
1	GO:0000050	BP	urea cycle	1/28	11/34644	0.008855861	1
1	GO:0071941	BP	nitrogen cycle metabolic process	1/28	11/34644	0.008855861	1
1	GO:0019627	BP	urea metabolic process	1/28	11/34644	0.008855861	1
3	GO:0043434	BP	response to peptide hormone	3/28	536/34644	0.009051284	1
1	GO:0007494	BP	midgut development	1/28	12/34644	0.009657179	1

1	GO:0009240	BP	isopentenyl diphosphate biosynthetic process	1/28	12/34644	0.009657179	1
1	GO:0046490	BP	isopentenyl diphosphate metabolic process	1/28	12/34644	0.009657179	1
1	GO:1904385	BP	cellular response to angiotensin	1/28	12/34644	0.009657179	1
12	GO:0048522	BP	positive regulation of cellular process	12/28	7426/34644	0.009975729	1
9	GO:0048583	BP	regulation of response to stimulus	9/28	4755/34644	0.010104694	1
1	GO:0006878	BP	cellular copper ion homeostasis	1/28	13/34644	0.010457872	1
1	GO:0055070	BP	copper ion homeostasis	1/28	16/34644	0.012856207	1
1	GO:0033591	BP	response to L-ascorbic acid	1/28	17/34644	0.013654406	1
1	GO:0010832	BP	negative regulation of myotube differentiation	1/28	17/34644	0.013654406	1
3	GO:1901652	BP	response to peptide	3/28	637/34644	0.014404009	1
1	GO:0006828	BP	manganese ion transport	1/28	18/34644	0.014451982	1
1	GO:0010310	BP	regulation of hydrogen peroxide metabolic process	1/28	18/34644	0.014451982	1
2	GO:0001889	BP	liver development	2/28	239/34644	0.015920774	1
1	GO:0006525	BP	arginine metabolic process	1/28	20/34644	0.01604527	1
2	GO:2000377	BP	regulation of reactive oxygen species metabolic process	2/28	242/34644	0.016299573	1
1	GO:0032026	BP	response to magnesium ion	1/28	21/34644	0.016840982	1
2	GO:1901215	BP	negative regulation of neuron death	2/28	254/34644	0.017853235	1
1	GO:0007340	BP	acrosome reaction	1/28	23/34644	0.018430545	1
1	GO:0009084	BP	glutamine family amino acid biosynthetic process	1/28	23/34644	0.018430545	1
1	GO:1990776	BP	response to angiotensin	1/28	25/34644	0.020017629	1
1	GO:0031954	BP	positive regulation of protein autophosphorylation	1/28	26/34644	0.020810243	1
1	GO:0045662	BP	negative regulation of myoblast differentiation	1/28	27/34644	0.021602239	1
1	GO:0070989	BP	oxidative demethylation	1/28	28/34644	0.022393617	1
1	GO:0051354	BP	negative regulation of oxidoreductase activity	1/28	30/34644	0.023974522	1
1	GO:0060416	BP	response to growth hormone	1/28	30/34644	0.023974522	1
1	GO:0006829	BP	zinc ion transport	1/28	30/34644	0.023974522	1
1	GO:0051193	BP	regulation of cofactor metabolic process	1/28	30/34644	0.023974522	1
1	GO:1901739	BP	regulation of myoblast fusion	1/28	31/34644	0.02476405	1
12	GO:0048518	BP	positive regulation of biological process	12/28	8280/34644	0.025571172	1
2	GO:0008202	BP	steroid metabolic process	2/28	308/34644	0.025577243	1
1	GO:0006591	BP	ornithine metabolic process	1/28	33/34644	0.026341258	1
2	GO:0070374	BP	positive regulation of ERK1 and ERK2 cascade	2/28	313/34644	0.026350636	1

1	GO:2000773	BP	negative regulation of cellular senescence	1/28	34/34644	0.02712894	1
2	GO:0007584	BP	response to nutrient	2/28	332/34644	0.029375486	1
1	GO:0007173	BP	epidermal growth factor receptor signaling pathway	1/28	37/34644	0.029488299	1
1	GO:0008535	BP	respiratory chain complex IV assembly	1/28	37/34644	0.029488299	1
1	GO:0061003	BP	positive regulation of dendritic spine morphogenesis	1/28	37/34644	0.029488299	1
10	GO:0007165	BP	signal transduction	10/28	6529/34644	0.02966413	1
1	GO:0051154	BP	negative regulation of striated muscle cell differentiation	1/28	40/34644	0.031842141	1
1	GO:0006123	BP	mitochondrial electron transport, cytochrome c to oxygen	1/28	41/34644	0.032625531	1
1	GO:0060142	BP	regulation of syncytium formation by plasma membrane fusion	1/28	42/34644	0.033408309	1
1	GO:0019646	BP	aerobic electron transport chain	1/28	42/34644	0.033408309	1
1	GO:1903427	BP	negative regulation of reactive oxygen species biosynthetic process	1/28	42/34644	0.033408309	1
1	GO:0010165	BP	response to X-ray	1/28	44/34644	0.034972034	1
1	GO:0038127	BP	ERBB signaling pathway	1/28	45/34644	0.035752982	1
1	GO:0051602	BP	response to electrical stimulus	1/28	45/34644	0.035752982	1
1	GO:0030282	BP	bone mineralization	1/28	47/34644	0.037313049	1
2	GO:1901214	BP	regulation of neuron death	2/28	379/34644	0.03741876	1
1	GO:0090344	BP	negative regulation of cell aging	1/28	50/34644	0.039648587	1
1	GO:0006695	BP	cholesterol biosynthetic process	1/28	52/34644	0.041202576	1
1	GO:0031952	BP	regulation of protein autophosphorylation	1/28	53/34644	0.041978661	1
2	GO:0070372	BP	regulation of ERK1 and ERK2 cascade	2/28	412/34644	0.043515761	1
1	GO:1902653	BP	secondary alcohol biosynthetic process	1/28	55/34644	0.043529013	1
1	GO:0032720	BP	negative regulation of tumor necrosis factor production	1/28	57/34644	0.045076947	1
1	GO:0051148	BP	negative regulation of muscle cell differentiation	1/28	58/34644	0.045850007	1
1	GO:0017004	BP	cytochrome complex assembly	1/28	58/34644	0.045850007	1
1	GO:0010288	BP	response to lead ion	1/28	58/34644	0.045850007	1
1	GO:0010043	BP	response to zinc ion	1/28	59/34644	0.046622465	1
1	GO:0008299	BP	isoprenoid biosynthetic process	1/28	59/34644	0.046622465	1
1	GO:2000772	BP	regulation of cellular senescence	1/28	59/34644	0.046622465	1
2	GO:0048732	BP	gland development	2/28	429/34644	0.046792967	1
1	GO:0034103	BP	regulation of tissue remodeling	1/28	60/34644	0.047394319	1

1	GO:1903556	BP	negative regulation of tumor necrosis factor superfamily cytokine production	1/28	60/34644	0.047394319	1
1	GO:0035634	BP	response to stilbenoid	1/28	61/34644	0.04816557	1
1	GO:0010830	BP	regulation of myotube differentiation	1/28	61/34644	0.04816557	1
1	GO:0050775	BP	positive regulation of dendrite morphogenesis	1/28	63/34644	0.049706267	1
1	GO:0031838	CC	haptoglobin-hemoglobin complex	1/28	7/34644	0.005644334	1
1	GO:0033162	CC	melanosome membrane	1/28	14/34644	0.011257941	1
1	GO:0090741	CC	pigment granule membrane	1/28	14/34644	0.011257941	1
1	GO:0043083	CC	synaptic cleft	1/28	20/34644	0.01604527	1
1	GO:0005913	CC	cell-cell adherens junction	1/28	25/34644	0.020017629	1
1	GO:0031362	CC	anchored component of external side of plasma membrane	1/28	26/34644	0.020810243	1
1	GO:0034364	CC	high-density lipoprotein particle	1/28	28/34644	0.022393617	1
1	GO:0031233	CC	intrinsic component of external side of plasma membrane	1/28	29/34644	0.023184378	1
1	GO:0072562	CC	blood microparticle	1/28	29/34644	0.023184378	1
1	GO:0030057	CC	desmosome	1/28	32/34644	0.025552962	1
1	GO:1990777	CC	lipoprotein particle	1/28	38/34644	0.030273526	1
1	GO:0034358	CC	plasma lipoprotein particle	1/28	38/34644	0.030273526	1
1	GO:0032994	CC	protein-lipid complex	1/28	44/34644	0.034972034	1
4	GO:0005576	CC	extracellular region	4/28	1603/34644	0.038581813	1
1	GO:0045277	CC	respiratory chain complex IV	1/28	56/34644	0.044303282	1
1	GO:0015453	MF	oxidoreduction-driven active transmembrane transporter activity	1/28	5/34644	0.004034809	1
1	GO:0004452	MF	isopentenyl-diphosphate delta-isomerase activity	1/28	10/34644	0.008053918	1
1	GO:0045545	MF	syndecan binding	1/28	12/34644	0.009657179	1
1	GO:0101020	MF	estrogen 16-alpha-hydroxylase activity	1/28	15/34644	0.012057386	1
1	GO:0050649	MF	testosterone 6-beta-hydroxylase activity	1/28	16/34644	0.012856207	1
1	GO:0042301	MF	phosphate ion binding	1/28	16/34644	0.012856207	1
1	GO:0008401	MF	retinoic acid 4-hydroxylase activity	1/28	20/34644	0.01604527	1
2	GO:0004252	MF	serine-type endopeptidase activity	2/28	256/34644	0.018118105	1
2	GO:0008236	MF	serine-type peptidase activity	2/28	277/34644	0.020999464	1
2	GO:0017171	MF	serine hydrolase activity	2/28	281/34644	0.021568727	1

2	GO:0016825	MF	hydrolase activity, acting on acid phosphorus-nitrogen bonds	2/28	281/34644	0.021568727	1
1	GO:0016863	MF	intramolecular oxidoreductase activity, transposing C=C bonds	1/28	47/34644	0.037313049	1
1	GO:0005044	MF	scavenger receptor activity	1/28	49/34644	0.038870682	1
1	GO:0043394	MF	proteoglycan binding	1/28	54/34644	0.04275414	1
1	GO:0032451	MF	demethylase activity	1/28	55/34644	0.043529013	1
3	GO:0004175	MF	endopeptidase activity	3/28	1008/34644	0.046895079	1
1	GO:0015002	MF	heme-copper terminal oxidase activity	1/28	63/34644	0.049706267	1
1	GO:0004129	MF	cytochrome-c oxidase activity	1/28	63/34644	0.049706267	1
1	GO:0016676	MF	oxidoreductase activity, acting on a heme group of donors, oxygen as acceptor	1/28	63/34644	0.049706267	1
